# Workplace Protections and Burnout Among Brazilian Frontline Health Care Professionals During the COVID-19 Pandemic

**DOI:** 10.3389/fpsyg.2022.880049

**Published:** 2022-04-26

**Authors:** Karina Pereira-Lima, Sonia Regina Loureiro, Isabella Lara Machado Silveira, José Alexandre Crippa, Jaime Eduardo Cecílio Hallak, Antonio Waldo Zuardi, Flávia de Lima Osório

**Affiliations:** ^1^Departamento de Psiquiatria, Universidade Federal de São Paulo, São Paulo, Brazil; ^2^Departamento de Neurociências e Ciências do Comportamento, Universidade de São Paulo, Ribeirão Preto, Brazil

**Keywords:** burnout, occupational stress, health personnel (MeSH), physicians, nurses, workplace, COVID-19

## Abstract

Health care workers from low- and middle-income countries have been playing a critical role in overcoming the challenges related to the COVID-19 pandemic; yet little is known about the relationship between workplace protections and wellbeing of Brazilian health care workers during the pandemic. This study aimed to evaluate whether Brazilian health care workers were satisfied with their workplace measures to protect their physical and mental health during the pandemic, and to assess the associations of such levels of satisfaction with indicators of burnout. Licensed Brazilian health care professionals were recruited *via* popular media between 5/19/2020 and 8/23/2020 to complete an online survey including questions about their demographic/professional characteristics, satisfaction with their workplace protective measures during the pandemic, and validated questionnaires assessing neuroticism, resilient coping, and symptoms of burnout. Most participants reported being dissatisfied with their workplace measures to protect their physical (516, 56.3%) and mental health (756, 82.5%). In multivariable analysis adjusted for personal and environmental factors, dissatisfaction with workplace physical health protections was significantly associated with higher levels of emotional exhaustion (*B* = 1.08, 95% CI = 0.47–1.69) and depersonalization (*B* = 0.61, 95% CI = 0.10–1.12), and dissatisfaction with workplace mental health protections significantly associated with higher levels emotional exhaustion (*B* = 1.17, 95% CI = 0.40–1.95). Efforts to improve both physical and mental health protective measures are critical to guarantee that health care workers continue to provide care at their maximum capacity.

## Introduction

Accounting for roughly 11% of all reported deaths from the coronavirus disease 2019 (COVID-19) worldwide (COVID-19 Data Explorer, 2022), Brazil has been particularly struck by the pandemic. Health care workers of all types have been playing a critical role in overcoming the challenges related to overwhelmed health care systems, political disputes, and increased risk for infection (Lancet and The Lancet, 2020; Nguyen et al., 2020).

Previous studies suggest that in addition to the stressors related to working under pandemic conditions, health care workers from low- and middle-income countries face additional challenges related to the lack of appropriate resources and workplace support to deal with a global healthcare crisis (Bong et al., 2020; Carter et al., 2020; Keller et al., 2020; Migisha et al., 2021). Conditions of high demands and limited resources, such as the beginning of the COVID-19 pandemic in Brazil and other low- and middle-income countries, are known to increase the risk for burnout and other poor wellbeing indicators among health care workers (Bakker and Demerouti, 2017; Preti et al., 2020; Barello et al., 2021; De Simone et al., 2021).

Given the well-established associations between health care workers’ distress symptoms with negative outcomes to both health care professionals and their patients (Hall et al., 2016; Dyrbye et al., 2017; Pereira-Lima et al., 2019; Montgomery et al., 2021), understanding the role of workplace measures to protect not only the physical, but also the mental health of health care workers during situations of high demands and limited resources is critical not only during the COVID-19 pandemic, but also moving forward.

While previous studies point to a high prevalence of indicators of low wellbeing among Brazilian health care professionals during the COVID-19 pandemic (Campos et al., 2021; Salvador et al., 2021), to the best of our knowledge, no studies assessed whether Brazilian health care professionals’ indicators of dissatisfaction with their workplace measures to protect their physical and mental health were associated with their symptoms of burnout.

Here, we evaluated whether Brazilian health care workers were dissatisfied with the measures adopted by their workplace to protect their physical and mental health during the early and most critical phase of the pandemic and assessed the associations of such indicators of dissatisfaction with symptoms of burnout among these professionals.

## Methods

### Participants

Licensed health care professionals working across all Brazilian states were recruited *via* popular media (social media, television, radio, and institutional advertising) between 5/19/2020 and 8/23/2020, period that corresponded to the first wave of the COVID-19 pandemic in Brazil. Eligibility criteria included being a licensed health care professional currently working in a health care setting providing care to COVID-19 patients. Professional categories included in the study were: (I) physicians; (II) nursing workers (nurses, nurse technicians/aids, and radiology technicians), and (III) other (i.e., dentists, nutritionists, occupational therapists, pharmacists, physical therapists, psychologists, social workers, and speech therapists). The Institutional Review Board of the Hospital das Clínicas da Faculdade de Medicina de Ribeirão Preto - Universidade de São Paulo approved the study and all participants provided their informed consent on the study online platform.

### Data Collection

All participants completed a secure online survey *via* REDCap. Identifiable data (i.e., name, e-mail, and workplace) were used to confirm participants’ eligibility *via* public data available at the correspondent professional board and later encrypted on the databases to protect participants’ privacy.

Burnout indicators were measured using the emotional exhaustion and depersonalization subscales of the Abbreviated Maslach Burnout Inventory (aMBI) (Riley et al., 2018), which is a shortened version of the Maslach Burnout Inventory (Maslach et al., 1997). Both the emotional exhaustion and the depersonalization subscales of the MBI include three items ranging from 0 (never) to 6 (everyday). Each subscale is scored by summing up its items, with scores higher than eight for exhaustion and higher than five for depersonalization indicating high levels of burnout (Riley et al., 2018).

In addition to the aMBI, the study online survey inquired about participants’ (I) demographic/professional characteristics (i.e., sex, age, professional category, and years of professional experience); (II) personal variables previously associated with mental health indicators among health professionals {i.e., neuroticism [NEO-FIVE Factor Inventory (NEO-FFI-R)] (Costa and McCrae, 1997) and coping style [Brief Resilient Coping Scale (BRCS)] (Sinclair and Wallston, 2004)]}; (III) infection-related concerns [i.e., “Are you concerned about being infected with COVID-19 and with its associated risk of death?” (Yes/No); “Are you concerned about the possibility of your family members being infected with COVID-19?” (Yes/No)”; “Do you feel that family members and close people have avoided contact with you because of your work?” (Yes/No)]; and (IV) satisfaction with their workplace physical and mental health protections: [i.e., “Are you satisfied with the measures adopted by your workplace to protect the physical health of health care professionals?” (Yes/No); and “Are you satisfied with the measures adopted by your workplace to protect the mental health of health care professionals?” (Yes/No)].

### Statistical Analyses

We calculated summary descriptive statistics for the full sample of health care professionals.

Differences in the prevalence of dissatisfaction with workplace protective measures and indicators of burnout among different professional categories (i.e., physicians, nursing workers, and others) were respectively assessed with χ^2^ and one-way ANOVA tests with Bonferroni *post hoc* correction.

We examined for the associations between dissatisfaction with workplace protections and burnout using multiple linear regression while accounting for all measured demographic, professional, personal, and infection-related concern variables. A two-sided *P* < 0.05 was considered statistically significant. All analyses were conducted using SPSS version 21 (IBM Corp.).

## Results

Nine hundred sixteen (60.2%) of the 1,522 health professionals who assessed the study platform completed the survey (Table 1). Of those, 730 (79.7%) were female, 376 (41%) were nursing workers, 275 (30%) physicians, and 265 (29%) other health care professionals (i.e., dentists, nutritionists, occupational therapists, pharmacists, psychologists, physiotherapists, and social workers). The average age of the participants was 35 years old (SD = 9), and on average, participating health care workers had 10 years of professional experience (SD = 9). Compared to national census data from non-participants (IBGE, 2000), health professionals participating in our study were more likely to be female (79.7 vs. 69.0%, *p* < 0.0001).

**TABLE 1:** Characteristics of participants (*N* = 916).

Characteristic	*N* (%) or Mean (SD)
Women, *N* (%)	730 (79.7)
Men, *N* (%)	186 (20.3)
Age, mean (SD)	35.2 (9.2)
Nursing workers, *N* (%)*	376 (41.0)
Physicians, *N* (%)	275 (30.0)
Other health care professionals, *N* (%)^#^	265 (28.9)

*Nursing workers included nurses, nursing technicians, and radiology technicians.

*^#^Other health care professionals included dentists, nutritionists, occupational therapists, pharmacists, psychologists, physiotherapists, and social workers.*

### Indicators of Burnout

A total of 336 (36.7%) participants screened positive for indicators of high emotional exhaustion (i.e., EE-aMBI ≥ 9), and 167 (18.2%) for indicators of high depersonalization (i.e., DP-aMBI ≥ 6), indicating a high prevalence of indicators of burnout among this population. Mean scores for emotional exhaustion and depersonalization were 7.1 (SD = 5.1) and 3.0 (SD = 3.8), respectively.

With respect to levels of emotional exhaustion across different professional categories, no statistically significant difference was found between physicians (mean = 7.2, SD = 4.8), nursing workers (mean = 7.2, SD = 5.4), and other types of health care professionals (mean = 7.1, SD = 5.1) (*F* = 0.65, df = 2, *p* = 0.52). In contrast, levels of depersonalization significantly differed across professional categories (*F* = 11.89, df = 2, *p* < 0.001), with physicians demonstrating significantly higher levels of depersonalization than nursing workers [3.8 (SD = 4.3) vs. 3.0 (SD = 3.8), adjusted *p* = 0.02], and other health care workers [3.8 (SD = 4.3) vs. 2.2 (SD = 3.0), adjusted *p* < 0.001]; and nursing workers showing significantly higher levels of depersonalization than other health care professionals [3.0 (SD = 3.8) vs. 2.2 (SD = 3.0), adjusted *p* = 0.03].

### Satisfaction With Workplace Protections

When inquired about whether they were satisfied with the measures adopted by their workplace to protect their physical and mental health during the COVID-19 pandemic, most health care professionals participating in the present study reported to be dissatisfied with their workplace measures to protect their physical (*N* = 516, 56.3%) and mental health (*N* = 756, 82.5%).

The frequency of dissatisfaction with both physical and mental health protective measures was high across all professional categories, with nursing workers presenting the highest prevalence of dissatisfaction with both physical and mental health protective measures, followed by physicians, and other health care professionals (Table 2).

**TABLE 2:** Health care professionals’ reports of satisfaction with their workplace measures to protect their physical and mental health during the COVID-19 pandemic.

Satisfaction with workplace protective measures, *N* (%)	Physicians (*N* = 275)	Nursing workers (*N* = 376)	Other health care professionals (*N* = 265)	Total (*N* = 916)	χ^2^ (*P*-value)
**Physical health protective measures**					
Satisfied	125 (45.5)	140 (37.2)	135 (50.9)	400 (43.7)	12.4 (0.002)
Dissatisfied	150 (54.5)	236 (62.8)*	130 (49.1)*	516 (56.3)	
**Mental health protective measures**					
Satisfied	47 (17.1)	51 (13.6)	62 (23.4)	160 (17.5)	10.5 (0.005)
Dissatisfied	228 (82.9)	325 (86.4)*	203 (76.6)*	756 (82.5)	

*Statistically significant difference between groups – compared to the group “other health care professionals”, nursing workers were significantly more likely to be dissatisfied with their workplace measures to protect their physical and mental health (p < 0.01). Nursing workers included nurses, nursing technicians, and radiology technicians. Other health care professionals included dentists, nutritionists, occupational therapists, pharmacists, psychologists, physiotherapists, and social workers.

### Associations Between Workplace Protections and Burnout Indicators

In all multivariable models for burnout indicators, dissatisfaction with workplace protections during the COVID-19 pandemic remained significantly associated with higher levels of burnout, even when accounting for well-established personal and environmental risks for these problems among health care professionals.

Higher levels of neuroticism, lower levels of resilient coping style, and dissatisfaction with both physical and mental health protective measures were significantly associated with higher emotional exhaustion in multivariable models (Table 3).

**TABLE 3:** Multivariable model of emotional exhaustion indicators among Brazilian frontline health care workers during the COVID-19 pandemic.

Variables	B (95% CI)	β	*P*-value
**Demographic and professional characteristics**			
Men	–0.02 (–0.72–0.68)	–0.002	0.957
Age	0.01 (–0.02–0.04)	0.02	0.510
Other health care professional	(Reference)	–	–
Physician	0.45 (–0.29–1.19)	0.04	0.233
Nursing worker	0.26 (–0.41–0.93)	0.03	0.448
**Personal characteristics**			
Neuroticism	0.27 (0.24–0.31)	0.48	**< 0.001**
Resilient coping	–0.13 (–0.22—0.04)	–0.09	**0.006**
**Infection-related concerns**			
Concern to be infected and death risk	0.10 (–0.65–0.86)	0.01	0.790
Concern with infecting family	–0.58 (–2.09–0.93)	–0.02	0.450
Family and close people avoiding contact	0.15 (–0.42–0.72)	0.02	0.603
**Dissatisfaction with workplace protections**			
Dissatisfaction with workplace physical health protection	1.08 (0.47–1.69)	0.11	**< 0.001**
Dissatisfaction with workplace mental health protection	1.17 (0.40–1.95)	0.09	**0.003**

*R^2^ = 0.33. Bold values indicates the statistical significance.*

Multivariable models for depersonalization included male sex, to be a physician or a nursing worker, higher levels of neuroticism, concerns with risk of infection and death, and dissatisfaction with physical health protective measures as variables significantly associated with higher levels of depersonalization (Table 4).

**TABLE 4:** Multivariable model of depersonalization indicators among Brazilian frontline health care workers during the COVID-19 pandemic.

Variables	B (95% CI)	β	*P*-value
**Demographic and professional characteristics**			
Men	0.96 (0.37–1.55)	0.10	**0.001**
Age	–0.02 (–0.05–0.004)	–0.05	0.099
Other health care professional	(Reference)	–	–
Physician	1.42 (0.79–2.04)	0.17	**< 0.001**
Nursing worker	0.68 (0.12–1.25)	0.09	**0.017**
**Personal characteristics**			
Neuroticism	0.14 (0.11–0.17)	0.32	**< 0.001**
Resilient coping	–0.04 (–0.11–0.04)	–0.03	0.370
**Infection-related concerns**			
Concern to be infected and death risk	–0.98 (–1.61—0.35)	–0.10	**0.002**
Concern with infecting family	–0.63 (–1.89–0.64)	–0.03	0.334
Family and close people avoiding contact	0.29 (–0.18–0.77)	0.04	0.233
**Dissatisfaction with workplace protections**			
Dissatisfaction with workplace physical health protection	0.61 (0.10–1.12)	0.08	**0.019**
Dissatisfaction with workplace mental health protection	0.40 (–0.25–1.05)	0.04	0.230

*R^2^ = 0.17. Bold values indicates the statistical significance.*

## Discussion

This study found that most Brazilian health care workers participating in the present study were dissatisfied with the measures adopted by their institutions to protect their physical and mental health during the COVID-19 pandemic, which was significantly associated with higher levels of burnout. Importantly, such associations remained significant even when accounting for well-established predictors of burnout among health care professionals.

In line with prior studies, higher levels of neuroticism (Patel et al., 2018) and lower levels of resilient coping style (Shoman et al., 2021) associated with higher levels of emotional exhaustion, while male sex (Purvanova and Muros, 2010), to be a physician or a nursing worker (Patel et al., 2018; Dall’Ora et al., 2020), higher levels of neuroticism (Patel et al., 2018), and concerns with risk of infection or death (Bashkin et al., 2021) associated with higher levels of depersonalization. The present study adds to these findings by demonstrating that dissatisfaction with institutional measures to protect health care professionals physical and mental health were strongly associated with higher levels of burnout, even after accounting for these factors.

This study was carried out during the early phase of the COVID-19 pandemic in Brazil, which was marked by a severe limitation of resources including the lack of available vaccines, insufficient number of hospital beds, wide-spread of false information about the disease on social media, lack of appropriate protective equipment, and a limited number of health care workers to attend the demands of a overwhelmed healthcare system (Fiocruz, 2021). Therefore, our results underscore the critical role of workplace measures to protect the wellbeing of health care workers during situations of increased demands and limited resources.

Previous research has suggested that the acute distress during the COVID-19 pandemic may transfer to longer-term mental health consequences (McGinty et al., 2020). Given the well-established association of health professional burnout with negative outcomes to both health care professionals and their patients (Wallace et al., 2009), our findings suggest that health care professionals’ dissatisfaction with their workplace protective measures is likely to have serious implications to patient care not only during this pandemic, but also in the long-term.

Limitations of the present study include its cross-sectional design and possible sample bias. Due to our convenience sample approach, it is possible that health care professionals with different levels of burnout and dissatisfaction with their workplace protective measures were more or less likely to choose to participate in the present study. In addition, even though all measures were taken to protect participants’ confidentiality, participants needed to fill out their email and name in order to certify that they were active health care workers, which might have also introduced bias in our results. Importantly, health care workers’ satisfaction with their workplace measures was measured through a questionnaire specifically designed for the present study, which limits the generalizability of our results. Additionally, it is possible that other personal and work-related factors not accounted by the present study could influence our results.

With estimates that the disturbances caused by the pandemic on the Brazilian and other low- and middle-income countries health care systems are likely to take a long time to be completely overcome, investments in increasing both physical protective measures and the access to evidence-based mental health care is imperative to improve both the wellbeing of health care workers and patient care. Further studies should focus on identifying specific workplace conditions associated with LAMIC health care worker’s wellbeing and satisfaction with their workplace protective measures.

## Data Availability Statement

The raw data supporting the conclusions of this article will be made available by the authors, without undue reservation.

## Ethics Statement

The studies involving human participants were reviewed and approved by the Hospital das Clínicas da Faculdade de Medicina de Ribeirão Preto – Universidade de São Paulo. The patients/participants provided their written informed consent to participate in this study.

## Author Contributions

SL and FO conceived the ideas and study design. SL, IS, JC, JH, AZ, and FO contributed to the acquisition of the data. KP-L, SL, and FO analyzed and interpreted the data. KP-L and FO drafted the manuscript. KP-L, SL, IS, JC, JH, AZ, and FO critically revised the manuscript for important intellectual content. FO obtained study funding. FO and SL supervised the study. All authors contributed to the article and approved the submitted version.

## Conflict of Interest

The authors declare that the research was conducted in the absence of any commercial or financial relationships that could be construed as a potential conflict of interest.

## Publisher’s Note

All claims expressed in this article are solely those of the authors and do not necessarily represent those of their affiliated organizations, or those of the publisher, the editors and the reviewers. Any product that may be evaluated in this article, or claim that may be made by its manufacturer, is not guaranteed or endorsed by the publisher.
